# Computational Modeling and Design of New Inhibitors of Carbapenemases: A Discussion from the EPIC Alliance Network

**DOI:** 10.3390/ijms23179746

**Published:** 2022-08-28

**Authors:** Elias Dahdouh, Lisa Allander, Linda Falgenhauer, Bogdan I. Iorga, Stefano Lorenzetti, Íñigo Marcos-Alcalde, Nathaniel I. Martin, Luis Martínez-Martínez, Jesús Mingorance, Thierry Naas, Joseph E. Rubin, Francesca Spyrakis, Thomas Tängdén, Paulino Gómez-Puertas

**Affiliations:** 1 Clinical Microbiology and Parasitology Department, Instituto de Investigación Sanitaria del Hospital, Universitario La Paz (IdiPAZ), 28046 Madrid, Spain; 2 Department of Medical Sciences, Uppsala University, 752 36 Uppsala, Sweden; 3 Institute of Hygiene and Environmental Medicine, Justus Liebig University Giessen, 35392 Giessen, Germany; 4 Institut de Chimie des Substances Naturelles (ICSN), CNRS UPR 2301, Université Paris-Saclay, 91190 Gif-sur-Yvette, France; 5 Department of Food Safety, Nutrition and Veterinary Public Health, Istituto Superiore di Sanità (ISS), 00161 Rome, Italy; 6 Molecular Modeling Group, Centro de Biología Molecular Severo Ochoa (CBMSO, CSIC-UAM), 28049 Madrid, Spain; 7 Biological Chemistry Group, Institute of Biology Leiden (IBL), Leiden University, Sylviusweg 72, 2333 BE Leiden, The Netherlands; 8 Unit of Microbiology, University Hospital Reina Sofía, 14004 Córdoba, Spain; 9 Department of Agricultural Chemistry, Edaphology and Microbiology, Maimonides Biomedical Research Institute of Cordoba (IMIBIC), 14004 Córdoba, Spain; 10 Department of Microbiology, Hôpital de Bicêtre, Université Paris-Saclay, 91190 Gif-sur-Yvette, France; 11 Department of Veterinary Microbiology, University of Saskatchewan at Saskatoon, Saskatoon, SK S7N 5A2, Canada; 12 Department of Drug Science and Technology, University of Turin, 10125 Torino, Italy

**Keywords:** inhibitors of carbapenemases, virtual screening, approach rationalization

## Abstract

The EPIC consortium brings together experts from a wide range of fields that include clinical, molecular and basic microbiology, infectious diseases, computational biology and chemistry, drug discovery and design, bioinformatics, biochemistry, biophysics, pharmacology, toxicology, veterinary sciences, environmental sciences, and epidemiology. The main question to be answered by the EPIC alliance is the following: “What is the best approach for data mining on carbapenemase inhibitors and how to translate this data into experiments?” From this forum, we propose that the scientific community think up new strategies to be followed for the discovery of new carbapenemase inhibitors, so that this process is efficient and capable of providing results in the shortest possible time and within acceptable time and economic costs.

Computational biology techniques, such as molecular dynamics simulations applied to the study of macromolecules and macromolecular interactions of biological nature, allow, in an increasingly precise way, to approach rationalization at the molecular level for the causes of different diseases. Using virtual modeling techniques, it is possible to understand why a change in a single amino acid could cause a change in the activity of an enzyme or a group of macromolecules in the case of diseases of genetic origin. Likewise, the interactions between viral proteins and different target proteins in the host cell or the functioning of bacterial proteins responsible for their pathogenicity can be modeled, leading to the design of new antimicrobial compounds.

Resistance to antimicrobial agents, especially to carbapenems, is an increasing global concern. A very common mechanism for carbapenem resistance is the selection of carbapenemase-producing organisms (i.e., organisms that produce enzymes that are able to break down carbapenems) [1]. One promising strategy to combat this phenomenon is the discovery of carbapenemase inhibitors through computational analyses. This approach requires expertise across a wide range of fields in order to properly define search parameters and lead to a targeted search strategy. It also highlights the importance of building a network to facilitate knowledge exchange between these fields and form a new synergistic collaboration that can achieve much more than each of its separate elements. 

**The Problem of Carbapenemases**. Carbapenems belong to the β-lactam family of antibiotics and are generally reserved as a last-line treatment for a wide range of bacterial infections. As other β-lactams, they are able to form an acyl–enzyme complex with penicillin binding proteins (PBPs), thus blocking their activity and resulting in the disruption of the bacterial cell wall. They have a common side chain (R1) on the C6 atom of the β-lactam ring and an R2 side chain on C2 that varies from one carbapenem to another. The most commonly used carbapenems are ertapenem, imipenem, meropenem, and doripenem, and they have been extensively used in recent decades due to their broad-spectrum activity and favorable pharmacokinetic/pharmacodynamic (PK/PD) parameters [2].

With the increased usage of these antimicrobial agents, resistance towards them through the production of carbapenemases, enzymes that can break down carbapenems by hydrolysis, has increased throughout the world [3]. Carbapenemases pose a great risk to patients since they confer resistance not only to carbapenems but also to most, if not all, other β-lactams, with the exception of monobactams. Carbapenemase-producing *Enterobacteriaceae* can also silently disseminate among hospitalized patients through intestinal colonization and produce outbreaks all around the world [4]. Additionally, high mortality rates have been reported for infections with carbapenemase-producing isolates in critically ill patients [5]. The spread of carbapenemase genes is also very concerning from a One Health perspective. In fact, they have been detected in environmental samples that include hospital wastewater [6]. This could allow them to disseminate into rivers and, consequently, to watered crops, especially in low- and middle-income countries [7]. Finally, carbapenemase genes have also been detected in various foodstuffs that could serve as a reservoir for carbapenemase-producing bacteria [8].

Carbapenemases are β-lactamases and are classified into four classes according to the Ambler Classification System [9]. More than 800 carbapenemases belonging to 64 families have been reported to date (some examples of the most prevalent carbapenemases, showing their similarity in size and general structure, although with a certain diversity in the active center, are displayed in Figure 1). Class A, C, and D carbapenemases are serine β-lactamases that share some sequence and structural similarities. They all have a catalytic serine in the active site and are able to form an acyl–enzyme intermediate while breaking down carbapenems. Class A carbapenemases generally have a narrow active site and are structurally similar to other non-carbapenemase members of class A β-lactamases. Their carbapenem-hydrolyzing mechanisms are mainly related to the conformational change of the active site that is able to accommodate the R1 side chain of carbapenems. Some of the most clinically significant class A carbapenemases include the *Klebsiella pneumoniae* carbapenemase (KPC), imipenem-hydrolyzing β-lactamase (IMI), and some types of Guiana extended-spectrum β-lactamase (GES). Class D carbapenemases have a generally weaker activity against carbapenems as compared with class A carbapenemases and have a narrow active site. They hydrolyze carbapenems in a similar manner to class A carbapenemases. Some of the most clinically relevant Class D carbapenemases are the oxacillinases OXA-23, OXA-24/40, OXA-48, and OXA-58. Class C carbapenemases can break down carbapenems after hosting them through their widened R2 sub-site that can accommodate their R2 side chain. These enzymes additionally hydrolyze a wide range of β-lactam antibiotics, including cefoxitin and cefotetan (the cephamycins). In general, class C carbapenemases require additional mechanisms in order to induce carbapenem resistance (such as the absence of certain porins and/or efflux pump overexpression). Their clinical significance in terms of carbapenem resistance is limited compared with the other carbapenemases, with the exception of *Acinetobacter*-derived cephalosporinase-68 (ADC-68) and cephamycin-hydrolyzing β-lactamase-10 (CMY-10).

Class B carbapenemases are different from the others in the sense that they are metallo-β-lactamases (MBLs), having one or two zinc ions in their binding site. These ions are fundamental for the hydrolysis mechanism of MBLs that do not require the formation of an acyl–enzyme. Their binding site architecture allows for the accommodation of the long R2 chain of carbapenems and for the formation of extensive non-covalent interactions with the zinc ions and the other residues lining the pocket. Some of the most clinically relevant types are the Verona integron-encoded metallo-β-lactamase (VIM), New Delhi metallo-β-lactamase (NDM), *Pseudomonas* imipenemase (IMP), Sao Paulo metallo-β-lactamase (SPM), German imipenemase (GIM), and Seoul imipenemase (SIM).

**Carbapenemase inhibitors: a promising new approach**. The increasing threat posed by carbapenemases has raised alarms among all major infectious disease societies and organizations. However, the development of new antimicrobial agents that might have activity against carbapenemase-producing organisms is almost non-existent due to the unique set of difficulties that pharmaceutical companies face in new antibiotic development. This has led several groups toward the exploration of alternative strategies that would help to overcome the resistance caused by carbapenemases. Though some approaches, such as combination therapies, have shown promise against certain carbapenemase-producing bacteria [10], one of the most promising approaches is the use of carbapenemase inhibitors. Carbapenemase inhibitors can potentiate carbapenems against carbapenemase-producing organisms and can be combined with other antimicrobial agents to achieve bacterial killing. Two of these combinations have already shown promise in treating infections caused by carbapenemase-producing *Enterobacteriaceae*. The first combination is ceftazidime–avibactam, which has shown success against KPC and OXA-48 producing organisms, and the second is meropenem–vaborbactam, which has shown success in treating KPC-producing organisms [11]. Recently, paradigm shifting strides have been made regarding the discovery and mode of action of carbapenemase inhibitors [12], including the recent discovery that MBL inhibitors do not necessarily require direct interaction with zinc for them to perform their action [13]. 

Traditionally, the approaches used for the discovery of new molecules involve several methods. One strategy is to screen crude extracts from natural substances for biological activity against organisms of interest, without knowledge of the mechanism of action. Another method, called chemical screening, is based on selecting substances from biological sources and performing exhaustive biochemical analyses without taking into account their potential to be used as treatments. These identified substances would then be deposited in large databases and screened for potential therapeutic properties at a later stage. A third approach, called target-oriented screening, relies on first selecting the biological target and then screening compounds for their activity based on their predicted interactions with the target. These discovery pipelines have yielded the vast majority of the drugs being used at the present time, including antibiotics. Although historically effective, these approaches are resource- and manpower-intensive, and are prone to “re-discovering” the same molecules due to the use of similar algorithms and databases, which is financially and intellectually inefficient and can impede progress. This has opened the way for the exploration of alternative approaches to drug discovery. One such approach is applying machine learning and complex algorithms for the virtual screening of possible compounds that can have the desired effects.

Virtual screening of carbapenemase inhibitors, using molecular docking techniques based on different sampling and filtering algorithms, together with molecular dynamics-based applications to study specific inhibitor/enzyme interaction systems, is a successful approach that has previously demonstrated its utility [14]. One of the many advantages of using traditional approaches to drug discovery in recent decades is the generation of extensive databases of molecules. The strides taken in computational analyses have opened the way to new methods and approaches that have a wide range of applications. These applications include the description of the dynamical properties of macromolecules (including proteins), providing structural interpretation of experimental data, and simulating interactions between molecules. This exciting approach is being applied to bacterial resistance where a recent study used molecular modeling in order to define interactions between the oxacillinase OXA-405 and β-lactams. Moreover, computational techniques based on the β-lactamases’ intrinsic dynamics have shown great potential for designing inhibitors that can go beyond the hypothesis-generating level and show promising experimental results. 

The advantages of the virtual screening approach include improved financial and technical efficiency, and facilitating the generation of in silico evidence to guide the focus of experimental efforts. Nevertheless, this approach is not without disadvantages. It requires a great deal of computing power and expertise, and can be too time-consuming if appropriate search parameters are not adjusted in advance to speed up the process without reducing the sensitivity and specificity of the process. In addition, the myriad of databases, potential targets, and search parameters to choose from can prove to be quite challenging. Moreover, the potential toxic effects of the molecules and unfavorable PK/PD parameters might result in a loss of several years of searching and experimentation, as would be the case with any other method. 

In this context, it is important to count on the joint efforts of research groups with expertise in different areas of knowledge, capable of collaborating not only to obtain new carbapenemase inhibitors but, above all, establishing a useful framework and strategy for working on this discovery. For this reason, the “EPIC Alliance” network (Alliance for the Exploration of Pipelines for Inhibitors of Carbapenemases) (*Joint Programming Initiative on Antimicrobial Resistance* https://www.jpiamr.eu/projects/aepic; Date: 1 May 2020) was created. In brief, the aim of the EPIC Alliance is to discuss and plan optimization strategies for the computational approach that would allow for an efficient search for promising molecules that can be taken beyond the hypothetical level and tested through lab experiments, animal models, and clinical trials.

The action route of the “EPIC Alliance” network is used to create a discussion forum for the exchange of ideas and knowledge to bridge the gaps between different research groups and develop the best strategy for discovering carbapenemase inhibitors. This forum will attempt to discover the best biochemical, molecular, and physical parameters to be used for the data mining of carbapenemase inhibitors; define which databases are most suitable for data mining; determine how to translate the computational findings into experiments; and determine the cost-efficiency of this strategy.

The main question to be answered by the EPIC Alliance is, therefore, the following: “What is the best approach for data mining on carbapenemase inhibitors and how to translate this data into experiments?”

Currently, the “EPIC Alliance” is a consortium formed by 12 members from 7 different countries: Spain (Elias Dahdouh (coordinator), Jesús Mingorance, Luis Martínez-Martínez, and Paulino Gómez-Puertas), Italy (Stefano Lorenzetti and Francesca Spyrakis), France (Thierry Naas and Bogdan I. Iorga), The Netherlands (Nathaniel Martin), Canada (Joseph E. Rubin), Sweden (Thomas Tängdén), and Germany (Linda Falgenhauer). The consortium brings together experts from a wide range of fields that include clinical, molecular and basic microbiology, infectious diseases, computational biology and chemistry, drug discovery and design, bioinformatics, biochemistry, biophysics, pharmacology, toxicology, veterinary sciences, environmental sciences, and epidemiology. Uniting experts from these different fields is expected to result in a synergistic collaboration that can tackle the discovery of carbapenemase inhibitors from different angles and increase the chance of bringing the potentially discovered molecules to the market.

From this forum, we propose that the scientific community think up new strategies to be followed for the discovery of new carbapenemase inhibitors, so that this process is efficient and capable of providing results in the shortest possible time and within acceptable time and economic costs. The discussion is to be established in five phases: (i) information exchange: sharing their expertise amongst different fields, both theoretical and experimental; (ii) choosing biochemical, molecular, and physical parameters, in addition to the algorithms, pipelines, and databases that are most suitable for data mining of carbapenemase inhibitors; (iii) designing in vitro and in vivo experiments, and clinical trials; (iv) preparing a feasibility study to determine whether the new approach is better than existing ones, as well as preparing a proof of concept; and (v) evaluating the preliminary results obtained in order to optimize and fine-tune the strategy.

The combined efforts of a wide range of experts in these types of approaches are expected to bear fruit in a unified strategy that is beneficial to the scientific community as well as the global fight against multi-drug resistant bacteria.

## Figures and Tables

**Figure 1:**
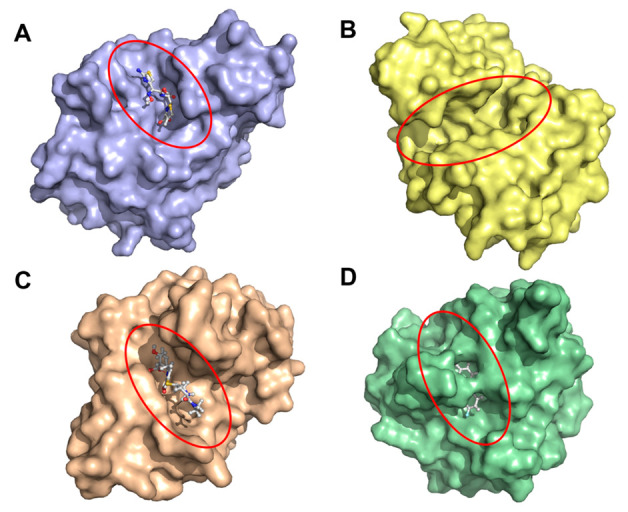
Molecular surface of representative β-lactamases, showing the position of the active site (red ovals). (**A**) KPC-2 enzyme (Protein Data Bank -PDB-: 5UJ3), with an inhibitor molecule located in the active site. (**B**) OXA-48 (PDB: 3HBR), showing the active site. (**C**) NDM-1 molecule (PDB: 4EYL), bound to hydrolyzed meropenem. (**D**) VIM-2 enzyme (PDB: 5ACX), showing the positions of some potential inhibitors.
